# Prospective UTE two-component MRI analysis of graft ligamentization after ACL reconstruction and association with demographic and surgical factors

**DOI:** 10.1186/s41747-025-00643-5

**Published:** 2025-10-31

**Authors:** Takeshi Fukuda, Akira Ogihara, Takenori Yonenaga, Daisuke Kubota, Hiroteru Hayashi, Ryuichi Itou, Hisashi Kitagawa, Katsutosi Murata, Stefan Sommer

**Affiliations:** 1 Department of Radiology, The Jikei University School of Medicine, Tokyo, Japan; 2 Department of Orthopaedic Surgery, The Jikei University School of Medicine, Tokyo, Japan; 3 Department of Radiology, The Jikei University Daisan Hospital, Tokyo, Japan; 4 Siemens Healthcare K.K, Tokyo, Japan; 5 Swiss Center for Musculoskeletal Imaging (SCMI), Balgrist Campus, Zurich, Switzerland; 6 Swiss Innovation Hub, Siemens Healthineers International AG, Zurich, Switzerland; 7 Swiss Innovation Hub, Siemens Healthineers International AG, Lausanne, Switzerland

**Keywords:** Anterior cruciate ligament, Anterior cruciate ligament reconstruction, Knee joint, Magnetic resonance imaging, Sports medicine

## Abstract

**Background:**

We aimed to evaluate longitudinal changes in ultrashort echo time (UTE) two-component biomarkers reflecting graft ligamentization after anterior cruciate ligament (ACL) reconstruction and to identify associated clinical factors.

**Materials and methods:**

Patients who underwent ACL reconstruction were prospectively included to perform 3-T three-dimensional double-echo UTE sequence at 3, 6, and 12 months postoperatively. Mean values of short T2* (T2*_s_), long T2* (T2*_l_), and fast fraction (FF), *i.e*., the signal proportion attributed to the T2*_s_ component, were calculated by fitting a biexponential model. Changes were analyzed using repeated measures analysis of variance−ANOVA. Multiple linear regression was used to assess associations between clinical factors and UTE parameters at 12 months.

**Results:**

Forty-two patients (20 males), aged 32.7 ± 15.0 years (mean ± standard deviation), were enrolled. T2*_s_ and T2*_l_ increased from 3 to 6 months (T2*_s_, 5.3 to 5.7 ms; *p* = 0.017; T2*_l_, 21.1 to 23.3 ms; *p* < 0.001), then decreased from 6 to 12 months (T2*_s_, 5.7 to 5.0 ms; T2*_l_, 23.3 to 21.1 ms; both *p* < 0.001). FF followed the opposite trend, decreasing from 0.29 to 0.25, then increasing to 0.30 (both *p* < 0.001). At 12 months, a higher body mass index (BMI) was associated with elevated T2*_s_ (*p* = 0.005), while semitendinosus-gracilis (STG) grafts (*p* = 0.018) and remnant preservation (*p* = 0.004) were associated with lower T2*_s_ values.

**Conclusion:**

UTE two-component analysis captures temporal changes in graft after ACL reconstruction, suggesting collagen regeneration. Higher BMI may hinder, while STG grafts and remnant preservation may promote ligamentization.

**Relevance statement:**

UTE two-component analysis serves as an imaging biomarker for ACL graft ligamentization, with higher BMI being associated with impaired ligamentization, while the use of STG grafts and remnant preservation may be associated with more favorable graft maturation at 12 months as assessed by UTE two-component MRI. These findings may help tailor rehabilitation protocols and guide graft selection.

**Trial registration:**

This study was prospectively registered with the University Hospital Medical Information Network Clinical Trials Registry (UMIN-CTR) under the identification number UMIN000045710 in October 2021.

**Key Points:**

- Ultrashort echo time two-component analysis noninvasively evaluates ligamentization of reconstructed ACL.
- Graft short T2* significantly decreased between 6 and 12 months postoperatively.
- Body mass index, graft type, and remnant status may influence graft maturation at 12 months.

**Graphical Abstract:**

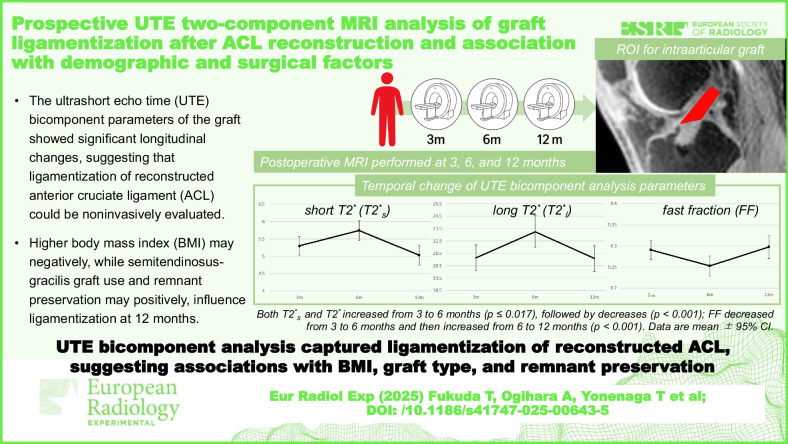

## Background

After anterior cruciate ligament (ACL) reconstruction, the graft undergoes a tissue transformation process called ligamentization, which involves both biological and mechanical changes. This process begins with acellular necrosis and inflammatory cell infiltration, followed by neovascularization and fibroblast proliferation [1, 2]. Over time, the graft remodels into a tissue composed of uniformly distributed, small-diameter collagen fibers. Assessing ligamentization is crucial for evaluating graft maturation and predicting long-term outcomes, including readiness to return to sports. While histological studies have provided valuable insights, human data are limited and show considerable inter-individual variability, differing from findings in animal models [2, 3]. As such, magnetic resonance imaging (MRI) has gained attention as a noninvasive tool to assess graft maturation on an individual basis.

In addition to the simple calculation of signal-to-noise ratio, several MRI techniques have been reported to assess graft maturation, including T2 mapping, diffusion tensor imaging, contrast enhancement, and ultrashort echo time (UTE) imaging [4–14]. UTE studies include two distinct analytical approaches. These are single-component analysis, which calculates a single T2* value from relatively fewer echo time (TE) images, and two-component analysis, which calculates two separate T2* components—short T2* (T2*_s_) and long T2* (T2*_l_). In addition, this analysis provides the fast fraction (FF), defined as the proportion of the T2*_s_ component relative to the total signal. Most studies have utilized single-component analysis, providing only a single T2*value, which incorporates various overlapping tissue changes. In ligament and tendon tissues, water exists in two main forms: bound water, which is tightly associated with collagen and proteoglycans, and free water, which is more mobile within the extracellular matrix. In UTE two-component analysis, T2*_s_ is thought to primarily reflect bound water and thus collagen organization, whereas T2*_l_ is more influenced by free water and is associated with tissue degeneration, necrosis, or increased vascularity. FF, which is the proportion of the T2*_s_ component, indirectly reflects the relative amount of bound water [15]. Recently, it has been demonstrated that the T2*_s_ do not correlate with T2* values obtained using the single component analysis, suggesting that T2*_s_ provides unique information about the structural quality of the grafts [16]. Furthermore, in studies on normal ACL, two-component analysis demonstrated superior fitting accuracy with lower root mean square error compared to single-component analysis [17]. Therefore, two-component analysis may be considered a more precise method for evaluating ligamentization.

Generally, elevated T2*_s_ values are considered indicative of deviation from normal collagen composition [18, 19], while lower FF indicates reduced bound water content and collagen disorganization. Therefore, if T2*_s_ values remain high at a stage where ligamentization has progressed to some extent, it may be considered an unfavorable condition. However, patient characteristics associated with elevated graft T2*_s_ values remain unclear.

We hypothesized that UTE two-component analysis parameters would show dynamic changes during the first postoperative year, reflecting the process of graft ligamentization. The novelty of this study lies in being the largest prospective cohort to date evaluating longitudinal changes with UTE two-component analysis up to 12 months after ACL reconstruction, and in examining their associations with clinical factors.

## Materials and methods

### Study population

This prospective study received our institutional review board approval (No. 32-312 10394), and written informed consent was obtained from all participants.

Patients who underwent ACL reconstruction between October 2021 and February 2024 were enrolled. Inclusion criteria were completion of research MRI sequences at 3, 6, and 12 months postoperatively. Exclusion criteria included missing MRI at any follow-up time point and severe image degradation due to motion or metallic artifact.

In addition to demographic data, the type of graft, the presence of meniscal injury, and whether the remnant was preserved during reconstruction were determined by reviewing intraoperative photographs and surgical records.

### MRI protocol

All MRI examinations were performed using a 3-T scanner (MAGNETOM Skyra, Siemens Healthineers). Clinical sequences included proton density-weighted imaging with Dixon technique, including in-phase, opposed-phase, water, and fat images, along sagittal and coronal planes. In addition, a three-dimensional double-echo UTE sequence was acquired with the following parameters: repetition time 70 ms; echo times (TEs) 0.04, 0.23, 0.47, 0.77, 1.18, 1.80, 2.88, 4.73, 7.07, 9.61, 12.26, and 15.0 ms; flip angle 16°; field of view 129 × 129 mm²; bandwidth 827 Hz/pixel; number of spokes 10,000; matrix size 128 × 128; slice thickness 1.2 mm with isotropic voxel; fat saturation applied; and total scan duration 23:40 min:s.

To minimize contamination of T2* measurements by the magic angle effect, all examinations were performed by trained technologists using a standardized protocol. A dedicated knee coil was used, and a positioning cushion was placed behind the knee to maintain a consistent degree of flexion and stabilize the limb during acquisition. For follow-up scans, the technologist reviewed the previous images of the same patient to reproduce the graft orientation relative to the B_0_ field as closely as possible.

### Image analysis

**Fig. 1:**
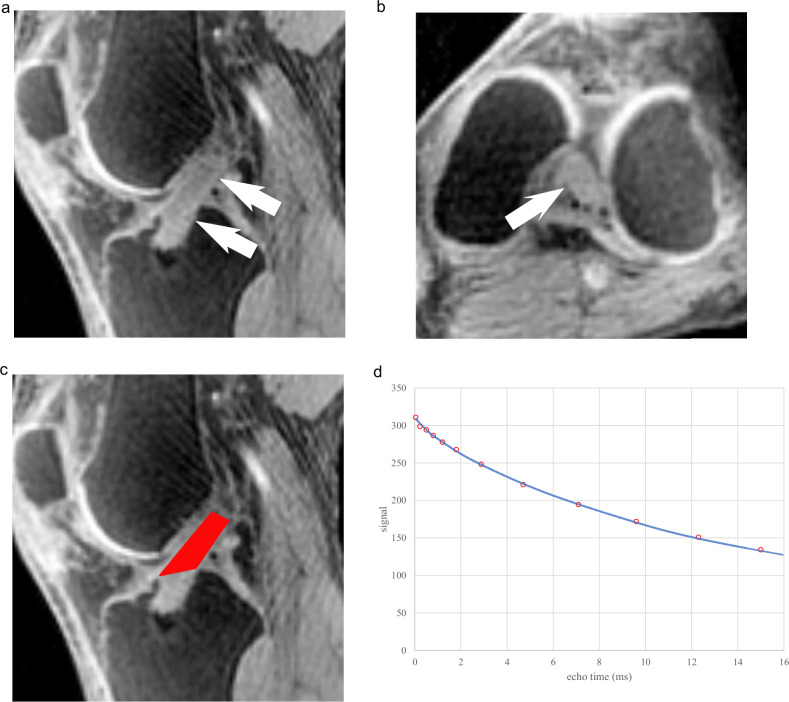
Representative ROI placement and signal fitting curve. **a** Sagittal first-echo ultrashort echo time (UTE) image (TE = 0.04 ms) showing the reconstructed anterior cruciate ligament (arrows) as a high-signal structure continuous from the tibial tunnel aperture. **b** Axial image showed the corresponding structure (arrow). **c** Final region of interest (ROI) displayed in red, encompassing the entire intra-articular portion of the graft between the tibial and femoral tunnel apertures, as defined in three dimensions using multiple imaging planes. **d** Biexponential fitting curve derived from signal decay within the ROI

MRI images were processed using MRIcron software (Rorden & Brett, 2000; https://www.nitrc.org/projects/mricron) and MATLAB (R2021a, MathWorks Inc.). Regions of interest (ROI) encompassing the intra-articular portion of the reconstructed ACL were manually defined by a musculoskeletal radiologist with 10 years of experience on the first-echo image (Fig. 1). The ROI was segmented as a three-dimensional volume encompassing the entire intra-articular portion of the graft, defined as the continuous high-signal structure extending from the tibial tunnel aperture to the femoral tunnel aperture. Segmentation was performed while viewing the sagittal, coronal, and axial planes simultaneously to ensure complete coverage of the graft and exclusion of adjacent structures. When necessary, clinical sequences such as proton density-weighted and fat-suppressed proton density-weighted images were referenced to aid in graft delineation. The ROI was delineated on the first-echo image (TE = 0.04 ms) to maximize SNR and conspicuity of the short T2 component, and verified on an intermediate echo (7th TE, 2.88 ms) where the short component had largely decayed, to avoid inclusion of synovial fluid and other adjacent structures to be excluded. To apply this ROI to all other TE images, all images from subsequent echoes were spatially registered with MATLAB to the first-echo images which the ROI was drawn. Inter-rater reproducibility was assessed by having a second radiologist, who is also a musculoskeletal radiologist with 16 years of experience, independently repeat ROI placement on a randomly selected subset of 20 patients. Both radiologists were blinded to all clinical information and to the ROI placement performed by the other reader during the analysis.

For two-component analysis, mean signal intensity within the ROI was analyzed using a biexponential model with Rician noise correction and nonlinear least-squares fitting:

$${\rm{S}}\left({\rm{TE}}\right)={\rm{FF}}* \exp \left(-\frac{{\rm{TE}}}{{T2}_{S}^{* }}\right)+\left(1-{\rm{FF}}\right)* \exp \left(-\frac{{\rm{TE}}}{{T2}_{l}^{* }}\right)$$

Where S is the signal; T2*_s_ is short T2*; T2*_l_ is long T2*; FF is the fast fraction, which is the fraction of the bound water.

### Statistical analysis

Normality of data distributions was evaluated using the Shapiro–Wilk test. Repeated measures analysis of variance (ANOVA) with Bonferroni *post hoc* correction was employed to compare temporal changes in calculated values (T2*_s_, T2*_l_, and FF) across follow-up periods (3, 6, and 12 months). Additionally, associations between these values at 12 months and clinical characteristics (age, sex, presence of meniscal injury, graft type, body mass index (BMI), and presence of remnant at the operation) were analyzed by multiple linear regression analysis. Inter-rater agreement of ROI placement was quantified using the Dice similarity coefficient. Statistical analyses were conducted using SPSS software (version 28.0, IBM Corp.), with statistical significance set at *p* < 0.05.

## Results

### Patients characteristics

Initially, 78 patients met the inclusion criteria, but 36 were excluded for the following reasons: 33 patients did not have imaging available at one or more time points, and 3 patients were deemed unevaluable due to artifacts. Ultimately, 42 patients were finally included: 20 males and 22 females, aged 32.7 ± 15.0 years (mean ± standard deviation), with a BMI of 22.8 ± 3.2 kg/m² (mean ± standard deviation). Meniscal injuries were present in 32 participants: medial meniscus (*n* = 19), lateral meniscus (*n* = 6), both menisci (*n* = 7). The graft types used were semitendinosus-gracilis (STG, *n* = 9), bone-tendon-bone (BTB, *n* = 18), and quadriceps tendon-patellar bone (QTB, *n* = 15).

### Temporal change of UTE biomarkers

**Fig. 2:**
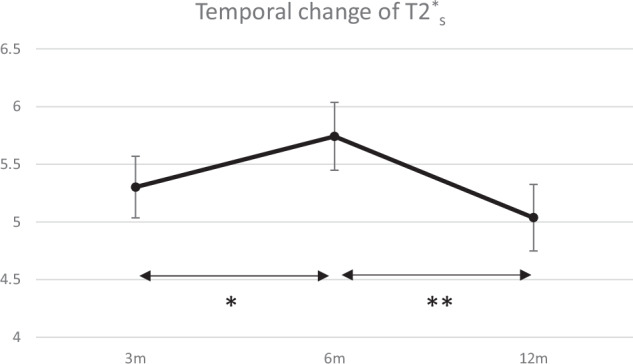
Temporal change of short T2* (T2*_s_). Data are presented as mean ± 95% confidence interval. * indicates a statistically significant increase from 3 to 6 months (*p* = 0.017). ** indicates a statistically significant decrease from 6 to 12 months (*p* < 0.001)

**Fig. 3:**
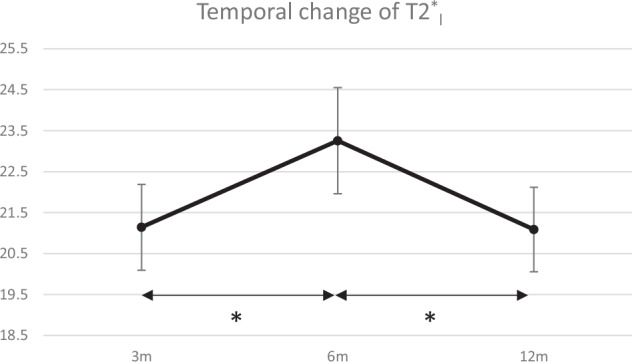
Temporal change of long T2* (T2*_l_). Data are presented as mean ± 95% confidence interval. * indicates a statistically significant increase from 3 to 6 months and a decrease from 6 to 12 months (*p* < 0.001)

**Fig. 4:**
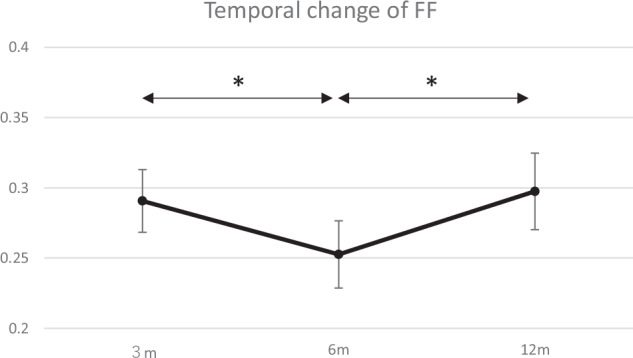
Temporal change of fast fraction (FF). Data are presented as mean ± 95% confidence interval. * indicates a statistically significant decrease from 3 to 6 months and an increase from 6 to 12 months (*p* < 0.001)

The mean T2*_s_ of the intra-articular graft at 3, 6, and 12 months postoperatively were 5.3 ± 1.1 ms, 5.7 ± 1.2 ms, and 5.0 ± 1.2 ms, respectively. There was a statistically significant increase from 3 to 6 months (*p* = 0.017), followed by a significant decrease from 6 to 12 months (*p* < 0.001) (Fig. 2). Similarly, the T2*_l_ exhibited the same pattern as the mean values of 21.1 ± 0.68 ms at 3 months, 23.3 ± 0.84 ms at 6 months, and 21.1 ± 0.67 ms at 12 months. There was a statistically significant increase from 3 to 6 months (*p* < 0.001), followed by a significant decrease from 6 to 12 months (*p* < 0.001) (Fig. 3). In contrast, the FF exhibited the opposite trend; at 3, 6, and 12 months, it was 0.29 ± 0.01, 0.25 ± 0.02, and 0.30 ± 0.02, respectively. It significantly decreased from 3 to 6 months (*p* < 0.001), followed by a significant increase from 6 to 12 months (*p* < 0.001) (Fig. 4).

### Association between clinical features and parameters at 12 months after surgery

Multiple linear regression analysis revealed that reconstruction using STG graft (B = -1.269, *p* = 0.018) and the presence of remnant tissue (B = -1.029, *p* = 0.04) were significantly associated with lower T2*_s_ at 12 months postoperatively (Table 1). On the other hand, high BMI was significantly associated with higher T2*_s_ (B = 0.168, *p* = 0.005). Other clinical variables, including age, sex, using BTB or QTB as the graft, and presence of meniscal injuries, were not significantly associated with low or high T2*_s_. BMI was also significantly associated with higher T2*_l_ (*B* = 0.796, *p* < 0.001) and lower FF (B = -0.019, *p* = 0.004) of the graft at 12 months postoperatively. All other clinical characteristics failed to show a significant relationship with high or low T2*_l_ and FF.

**Table 1:** Multivariate linear regression analysis for predictors of bicomponent analysis values at 12 months postoperatively

	T2^*^_s_		T2^*^_l_		FF	
	B	*p*-value	B	*p*-value	B	*p*-value
Age	0.007	0.588	0.068	0.194	-0.001	0.676
Gender	-0.059	0.879	0.296	0.845	0.015	0.725
BMI	0.168	**0.005**	0.796	**< 0.001**	-0.019	**0.004**
Use of STG	-1.269	**0.018**	-1.231	0.539	0.063	0.257
Use of BTB	0.268	0.485	0.493	0.72	-0.016	0.666
Use of QTB	-0.468	0.404	-2.608	0.235	0.035	0.564
Presence of meniscus injury	0.344	0.429	-1.041	0.538	0.03	0.523
Presence of remnant	-1.029	**0.04**	-2.113	0.267	0.078	0.145

Bold values indicate statistically significant associations

The regression coefficient (B) represents the direction and magnitude of the association between each independent variable and the bicomponent analysis values. A negative B value indicates a tendency toward lower values for that variable

*STG* Semitendinosus-gracilis graft, *BTB* Bone–patellar tendon–bone graft, *BMI* body mass index, *QTB* Quadriceps tendon-patellar bone graft

### Inter-reader reliability for ROI

Regarding the reproducibility of the ROI placement, the inter-rater Dice similarity coefficient was 0.74 (range: 0.68–0.83).

## Discussion

This study demonstrated that T2*_s_, which is associated with collagen composition, significantly increased from 3 to 6 months and subsequently decreased from 6 to 12 months. T2*_l_ also exhibited a similar pattern with a significant difference.

UTE two-component analysis allows for the separate quantification of T2*_s_ and T2*_l_, which are considered to reflect bound water and free water, respectively [18, 20]. The prolongation of T2*_s_ can be caused by subtle structural changes, such as disruption of collagen fiber alignment. A study using 7-T MRI reported that T2*_s_ in a degenerated Achilles tendon was nearly twice as long as that in a healthy tendon [21]. The significant increase in T2*_s_ observed between 3 and 6 months postoperatively in this study is likely to reflect collagen breakdown during early ligamentization. Given that this increase was accompanied by a significant rise in T2*_l_ and a decrease in FF, it may reflect a combination of necrosis, neovascularization, and inflammatory cell infiltration, all of which contribute to elevated free water content in the graft tissue [2, 3, 22, 23]. Based on the limited histological reports of human ligamentization, during the period corresponding to 3–6 months postoperatively, the proportion of mature collagen transiently decreases, and collagen fiber orientation tends to become irregular and disorganized. In addition, an increase in fibroblasts and marked neovascularization has been reported, followed by a gradual decline over time [2, 3, 24]. These processes are likely to increase interstitial water, thereby prolonging T2*_l_ and contributing to the concurrent rise in T2*_s_ due to collagen disorganization.

In contrast, the trend reversal observed between 6 and 12 months postoperatively—characterized by decreasing T2*_s_ and T2*_l_ and increasing FF—likely represents a transition toward the remodeling and maturation phases of ligamentization. Human biopsy studies generally demonstrated persistent hypercellularity relative to the native ACL, accompanied by cellular maturation and progressively improved alignment along the principal stress direction. Several human series have reported that collagen fiber orientation may remain irregular up to 12 months, despite macroscopic normalization of graft appearance [2, 3, 22–24]. Vascularity typically regresses from its early peak but may still be elevated compared with native tissue [3, 24]. Overall, these findings suggest that grafts undergo ongoing remodeling with incomplete ligamentization, characterized by persistent hypercellularity, declining yet persistent neovascularity, and gradual collagen reorganization rather than complete restoration of native-like architecture [25]. These structural and compositional changes would be expected to reduce free water content and restore bound water characteristics, reflected in the decreasing trend of T2*_s_ and T2*_l_ values, although these values still remained higher than those of native ligaments or tendons at 12 months postoperatively.

Previously, one study utilized an abbreviated two-component analysis protocol with a small number of patients following ACL reconstruction, focusing only on the 3- to 6-month postoperative period [14]. They reported no significant differences in their measured values during this time frame. However, their abbreviated protocol, which derived values from a limited number of TEs, potentially reduces the accuracy of the biexponential fitting. In addition, because they fixed the T2*_l_ value at 20 ms rather than measuring it, direct comparison with the present study, which measured both T2*_s_ and T2*_l_, is difficult. They also acknowledged this limitation and stated that future studies measuring both T2*_s_ and T2*_l_ components *in vivo* would provide a more complete characterization of graft water compartments during ligamentization. In contrast, our study employed a greater number of TEs, ensuring more robust quantification of T2*_s_ and T2*_l_ components. Moreover, our study included a larger patient cohort with an extended follow-up period of 12 months, which provides a more comprehensive understanding of the dynamic changes in ligamentization. Other studies have also evaluated temporal changes in graft T2* values using single-component analysis [9, 12, 13]; however, the parameters obtained with this approach are different from those derived from two-component analysis, making meaningful comparison difficult.

The factors that promote successful ligamentization remain poorly understood. Given that normal ligament tissue is characterized by short T2*_s_ and T2*_l_ values as well as a high FF, it is reasonable to assume that reconstructed grafts will gradually progress toward these values over time. In this study, at the final time point of 12 months postoperatively, higher BMI was significantly associated with suboptimal trends across all parameters, which is higher T2*_s_ and T2*_l_ and lower FF. It may suggest that elevated BMI may hinder the maturation of the graft. The association between BMI and graft reinjury remains inconsistent. A previous study that followed 30,591 patients who underwent ACL reconstruction for two years reported that patients with a BMI of 25 or higher had an increased risk of graft failure [26]. They discussed that elevated BMI may lead to increased mechanical load on the knee joint and may delay muscle recovery and the acquisition of joint stability due to suboptimal rehabilitation progress. On the other hand, there is a study that reported a higher BMI in younger individuals is associated with a decreased risk of revision surgery [27]. In that report, it was hypothesized that the lower risk of reinjury in patients with high BMI was attributable to lower levels of physical activity.

Our cohort was recruited from a sports medicine clinic outpatient, and many of the patients engaged in daily physical activity regardless of their BMI. These differences in patient characteristics may explain the discrepancy between our findings and this previous study. These findings may provide insight into potential factors influencing graft maturation as assessed by MRI. In patients with elevated BMI, rehabilitation protocols may need to be individualized in terms of exercise progression and by scheduling more frequent follow-ups to monitor graft maturation and avoid risks of delayed recovery or reinjury.

Interestingly, the use of an STG graft and the presence of remnant tissue were significantly associated with lower T2*_s_ values at 12 months postoperatively in this study. Notably, a previous systematic review also reported that grafts using hamstring tendons, particularly with remnant preservation, tend to exhibit lower MRI signal intensities within the first year postoperatively compared to BTB grafts, indicating a higher degree of graft maturation [28]. These observations are consistent with our results and further support the notion that hamstring autografts, especially with remnant preservation, may facilitate more favorable biological healing within the first year after ACL reconstruction. These observations may have practical implications, suggesting that when patient-specific factors permit, selecting an STG autograft and preserving remnant tissue could favor earlier graft maturation during the first postoperative year. However, evidence on the superiority of one graft type over another is still unclear [29, 30], and MRI signal-based maturity should be interpreted cautiously and in conjunction with functional criteria and reinjury risk assessment.

The term “remnant” refers to the remaining tissue of the torn ACL after injury. This residual tissue often includes parts of the torn ligament still attached to the tibia or femur, and it may retain biological elements such as cells, blood vessels, synovium, and mechanoreceptors [31, 32]. Although current evidence does not conclusively demonstrate that remnant preservation significantly enhances graft ligamentization, recent studies have highlighted its potential histological advantages [31, 33, 34]. The remnant tissue often contains key biological components—such as synovial membrane, vascular structures, and mechanoreceptors—that may theoretically promote revascularization, synovial coverage, and proprioceptive recovery, all of which are essential for effective graft maturation. Based on this theoretical framework, remnant-preserving techniques have increasingly been adopted in ACL reconstruction. In our study, we observed a tendency toward lower T2*_s_ values at 12 months postoperatively in cases with remnant preservation, suggesting more advanced graft maturation compared to those without a remnant.

This study has several limitations. First, although histological evaluation would provide definitive evidence regarding ligamentization, such invasive assessment is ethically unfeasible in clinical human studies. Second, because we aimed to evaluate longitudinal changes over a 1-year period with scheduled MRI scans, increasing the sample size was not easily achievable. Nearly half of the originally enrolled patients were excluded, mainly due to incomplete imaging at all scheduled time points. This exclusion may have introduced selection bias, as the analyzed cohort may represent patients with higher compliance with follow-up and better rehabilitation adherence, potentially limiting the generalizability of our findings. In addition, given that three different graft types were included in the analysis, a larger sample size would be desirable to enhance statistical power. This study has a pilot nature as a prospective observational study, and therefore, no *a priori* power calculation was performed. However, it included consecutive patients who underwent ACL reconstruction during the study period, and the sample size was larger than in most previous reports on UTE two-component analysis of ACL grafts. Third, two-component analysis requires the acquisition of multiple TE images. In this study, we used 12 TE points, which may be considered modest. Although a greater number of TEs could potentially improve the accuracy of biexponential fitting, increasing the number of echo times would result in a longer scan duration. In our institution, acquiring 12 TE images was considered the feasible maximum without compromising patient compliance. The protocol used in this study required approximately 23 min of scan time because only two TE images could be obtained per repetition time. If multiple echoes could be acquired within a single repetition time, scan time could be substantially reduced, potentially facilitating clinical implementation. Fourth, our cohort consisted only of patients with uneventful postoperative courses, and thus, did not include cases of graft re-rupture or other complications. Fifth, the single-center nature of our study may restrict the generalizability of the findings to broader populations. Finally, while our follow-up period extended to 12 months, some studies suggest that graft ligamentization in humans may continue for 2 to 3 years [3]. Therefore, a longer follow-up would be necessary to fully capture the entire course of ligamentization. We are currently continuing to follow this patient cohort, and future investigations with extended follow-up are planned to provide a more comprehensive understanding of the ligamentization process over time. In addition, the current study did not include functional outcome measures, but given that all patients in our cohort completed one year without complications, substantial differences in functional outcomes might not have been observed at this time point. However, should graft re-ruptures or other adverse events occur in the future, re-evaluating the current imaging findings in relation to functional outcomes would be of interest. Future studies incorporating such functional measures, along with investigating faster acquisition techniques, could further clarify the clinical relevance of UTE two-component analysis and facilitate its transition into routine practice.

In conclusion, this study demonstrated that UTE two-component analysis captures distinct temporal changes in graft tissue following ACL reconstruction. In particular, a significant decrease in T2*_s_ and T2*_l_ from 6 to 12 months postoperatively, along with an increase in FF, may suggest progressive collagen reorganization. Furthermore, higher BMI may be associated with impaired ligamentization, while the use of STG grafts and remnant preservation may be associated with more favorable graft maturation at 12 months as assessed by UTE two-component MRI.

## Data Availability

The datasets generated and/or analyzed during the current study are not publicly available due to restrictions related to privacy or ethical concerns, but are available from the corresponding author on reasonable request.

## Abbreviations

ACL: Anterior cruciate ligament
BMI: Body mass index
BTB: Bone-tendon-bone
FF: Fast fraction
QTB: Quadriceps tendon-patellar bone
ROI: Region of interest
STG: Semitendinosus-gracilis
T2*_l_: Long T2*
T2*_s_: Short T2*
TE: Echo time
UTE: Ultrashort echo time
